# Review of biomimetic ordered microstructures in advancing synergistic integration of adhesion and microfluidics

**DOI:** 10.1039/d3ra07698a

**Published:** 2024-04-10

**Authors:** Meng Wei, Qian Zhou, Xiaoming Ma, Bingbing Gao

**Affiliations:** a School of Pharmaceutical Sciences, Nanjing Tech University Nanjing 211816 China gaobb@njtech.edu.cn; b Department of Orthopedics, Taizhou People's Hospital 366 Taihu Road Taizhou Jiangsu Province People's Republic of China 1229917328@qq.com

## Abstract

Many ordered arrangements are observable in the natural world, serving not only as pleasing aesthetics but also as functional improvements. These structured arrangements streamline cohesion while also facilitating the spontaneous drainage of liquids in microfluidics, resulting in effective separation and signal enhancement. Nevertheless, there is a substantial challenge when handling microstructured chips with microfluidic detection and adhesion. The arrangement of the adhesive interface's microstructure affects the liquid flow in the microfluidic chip, impacting the detection's sensitivity and accuracy. Additionally, the liquid in the microfluidic chip corrodes the adhesive material and structure, reducing the adhesion strength due to the hydration layer between the material and the contact interface. Therefore, this review explores the application of ordered structures in the integration of adhesion and microfluidics. We discussed the standard preparation method, appropriate materials, and the application of ordered structures in biomimetic adhesion and microfluidics. Furthermore, the paper discusses the major challenges in this field and provides opinions on its future developments.

## Abstract 2

Many ordered arrangements are observable in the natural world, serving not only as pleasing aesthetics but also as functional improvements.

## Graphical abstract

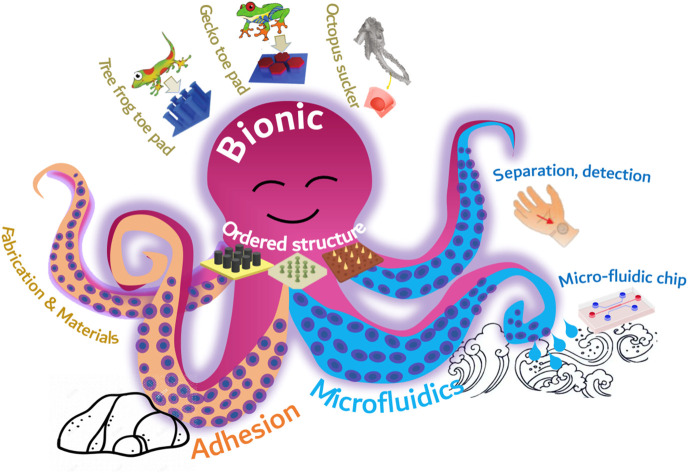

## 1. Introduction

As individuals become increasingly health-conscious, a growing number of health testing devices have become available for purchase. These range from highly precise and comprehensive instruments that offer intricate and precise analysis to simple daily wearable electronic watches that only provide limited measurements.^1–3^ However, the limitations of inaccurate and inadequate electronic testing devices require users to seek professional testing, resulting in a significant investment of time and energy. This is contrary to the current objective of immediate and expert health assessment. Therefore, a professional measurement and portable health monitoring device is necessary.

Recently, there has been growing interest in employing microfluidic technology as a portable method for professional detection.^4–6^ Developed from the Micro-Electro-Mechanical System (MEMS), microfluidic technology enables precise manipulation of minuscule amounts of fluid in microtubules at the micron scale.^7–9^ Its primary aim is to condense biological, chemical, and other laboratories into a chip measuring just a few square centimeters.^10,11^ It offers features such as sample preparation, reaction, separation, and detection.^12–17^ Microfluidic chips are frequently utilized for point-of-care testing (POCT), permitting the decoding of human health data with the assistance of bodily fluids.^18–20^ They are professional and reliable, but fixation typically relies on external forces. The most common method of fixation is using a double-sided adhesive film, which is usually irreversible and causes pain when removed. Moreover, its low biocompatibility also poses difficulties for users. The adhesion of the microfluidic chip to the joint area that moves is hindered by the binding of external tape. However, the uptake and detection of bodily fluids will also be affected by the adhesion of the microfluidic chip itself, whether through a double-sided film or hydrogel. Therefore, an adhesive method that can achieve strong adherence throughout the entire human body without impacting microfluidic chip performance is needed. Geckos can adhere robustly to both rough and wet vertical surfaces due to the ordered microstructure arrangement of their toes.^21–23^ This enables them to maintain adhesion without the utilization of any adhesives. The development of new materials or devices by imitating the components, structures, or functions of natural organisms is commonly known as “bionicsˮ.^24–32^ By replicating the adsorption microstructures found on gecko toe pads, tree frog and octopus suckers, researchers have achieved adhesion on a range of complicated surfaces.^33–38^ This adhesive method boasts the revolutionary benefits of easy detachment and excellent biocompatibility.^39,40^ During the process of surface switching adhesion, the microfluidic channel remains stable, while only the adhesion microstructure undergoes alteration.^28,41,42^ This innovative method of adhesion enables the patch to display a dependable and steady adhesive force on the skin's surface, even in humid conditions.^43–45^ Moreover, it can be effortlessly detached from the skin without inducing substantial discomfort.

**Fig. 1:**
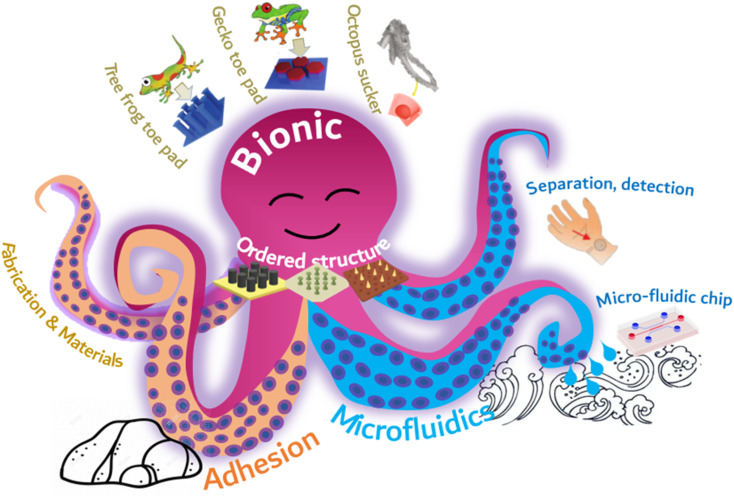
Schematic illustration of the types of ordered structures, biomimetic adhesion, and microfluidics.

By mimicking the arranged microstructure of gecko toe pads and octopus suckers, it is possible to achieve robust adhesion without compromising the functionality of microfluidic chips. Nevertheless, there remain some obstacles in the preparation of chips with both adhesion and microfluidic capabilities. The industrial production of ordered adherable microfluidics faces major challenges, namely, complex manufacturing, difficult integration, and high production costs. Inadequate design of the adhesion structure has an impact on liquid flow within the flow control channel, leading to a decrease in detection sensitivity. Furthermore, the liquid properties of the surface tissue gradually wear down and destabilize the microstructural materials affixed to it. In this paper, we initially present commonly used adhesion materials and processing techniques, with particular emphasis on bionic adhesion *via* ordered microfluidic processing methods. This is followed by an extensive account of diverse ordered microstructures, which utilize bionic ordered structures to achieve adhesion to an array of surfaces, including those with spilled liquid, sweat skin surfaces, and reversible adhesion that can be torn. Simultaneously, constructing microfluidics in an ordered structure enables real-time liquid monitoring. Subsequently, a concise overview of the integrated application and future development prospects of biomimetic adhesion ordered microfluidics is provided (Fig. 1).

## 2. Fabrication and materials

As healthcare has received increasing attention, interest in real-time body monitoring has grown significantly. However, individuals presently depend on bulky and complex equipment for health assessment, which is generally only available in professional healthcare environments. This exacerbates the already challenging process of testing. The introduction of microfluidic chips provides a plausible solution to this problem. Microfluidic technology can instantly evaluate human health by analyzing the components in bodily fluids. This technology examines bodily fluids to assess human health status. It has the capacity to provide a fast and accurate analysis of human health. Currently, reports have stated that microfluidic stickers for human health monitoring use the analysis of sweat and wound secretions on the body's surface. These stickers, however, typically require external forces for adherence, exhibit poor adhesion to wet and sweaty skin, and cannot accurately adhere to unhealed wounds. As a result, the results of using these microfluidic stickers are often inaccurate, and removing them causes more pain for patients with wounds. Hence, the development of a microfluidic chip with adhesive capabilities is crucial. Nonetheless, conventional adhesives utilize double-sided adhesive tape, impeding human secretions from effectively entering the microfluidic channel. Furthermore, the chemical components of the adhesive film exhibit low biocompatibility. They often lead to difficult tearing, poor adhesion to rough and wet surfaces, and long-term use easily results in skin discomfort. This clearly contradicts the initial purpose of designing microfluidic chips for extended wear and real-time detection. Fortunately, it was observed that the gecko could securely climb vertical walls without the need for adhesive due to the microscopic structure of the bristles on its claws. Octopus suction cups, aided by a specialized cup structure, can tightly adsorb to different surfaces by exploiting the pressure difference between their internal and external surfaces. Mosquito mouthparts can penetrate an animal's body undetected due to their incredibly fine, needle-like structure. Our proposal is to improve the adhesive patch's adhesion ability and ensure that the microfluidic chip operates normally by utilizing a specific microstructure, inspired by this biological phenomenon. In the current research, adhesive patches inspired by biology display qualities such as robust adhesiveness, easy detachment, and high biocompatibility. The design of microscopic adhesion structures often employs polyurethane (PU) and polydimethylsiloxane (PDMS), with a prevalent use of the template method.^49^

### 2.1. Biomimetics design

Geckos are able to crawl freely on walls due to the hydrophobic performance and reversible adhesion of their feet. Geckos are able to crawl freely on walls due to the hydrophobic performance and reversible adhesion of their feet. The multi-scale micro-nano structure at the tip of their toes is responsible for this adhesion, consisting of almost 500 000 bristles with a length ranging from 30 to 130 μm and a diameter of 5 μm. The multi-scale micro-nano structure at the tip of their toes is responsible for this adhesion, consisting of almost 500 000 bristles with a length ranging from 30 to 130 μm and a diameter of 5 μm. At the end of each bristle, there are numerous hairs with a diameter and length ranging from 200 to 500 nanometers as shown in Fig. 1a. When numerous nanostructured hairs come into contact with a surface, van der Waals forces accumulate and provide enough support for geckos to walk on walls.

Researchers attempt to simulate this microstructure using methods and materials to achieve similar adhesion abilities to gecko feet. Currently, polymer materials are primarily used to prepare imitation gecko foot adhesion materials. Researchers at the University of Manchester have successfully obtained a polyimide cilia array measuring 50 μm × 50 μm, with a diameter of 200–400 nm, a length of 150–200 nm, and a spacing of 400–450 nm. The cilia were produced using oxygen plasma dry etching combined with electron beam etching. However, the adhesion ability of the cilia is limited due to their tendency to adhere to each other and reduce the contact area with the base. Subsequent researchers have continuously improved this model using various methods. One such method involves using porous anodic alumina (PAA) as a template, prepared by mild anodizing, and coating the PAA template with polyimide prepolymer through spinning coating. The resulting polyimide (PI) array film exhibits good adhesion to water as shown in Fig. 1b.

### 2.2. PDMS

**Fig. 2:**
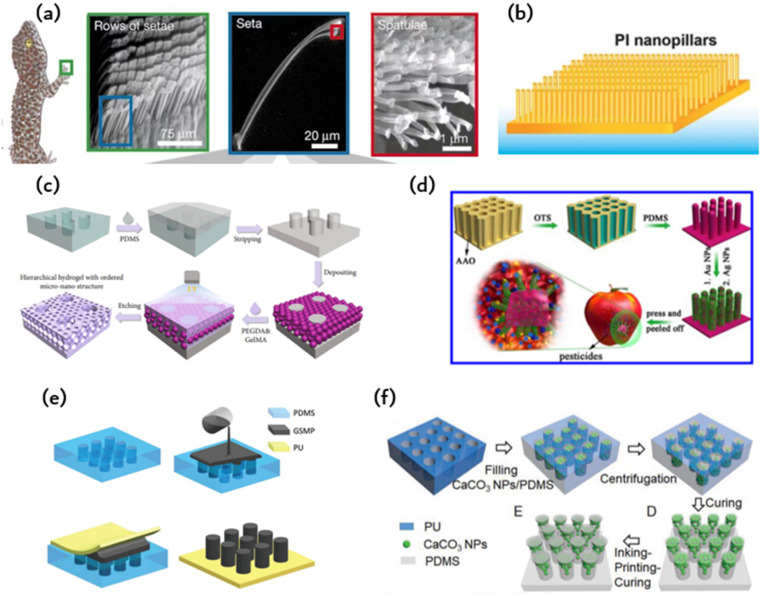
The material and fabrication method of bioinspired adhesion: (a) microscopic view of the bristles on the toe pads of a gecko;^46^ Reprinted with permission. Copyright 2000 Macmillan Magazines Ltd. (b) Polyimide microcolumns resembling gecko bristles.^33^ (c) Schematic of the preparation of the hierarchical hydrogel with an ordered micronano structure using polyethylene glycol diacrylate (PEGDA) and methacrylate gelatin (GelMA) as materials by a polydimethylsiloxane (PDMS) template;^47^ Reprinted with permission. Copyright 2021 Luyao Zhu *et al.* Exclusive Licensee Science and Technology Review Publishing House. (d) Schematic of the PDMS film constructed by an anodic aluminum oxide (AAO) template;^48^ Reprinted with permission. Copyright 2017 American Chemical Society. (e) Schematic of the fabrication of polyurethane (PU)-graphene/shape memory polymer (GSMP) by mold casting;^24^ Reprinted with permission. Copyright 2019 Wiley-VCH Verlag GmbH & Co. KGaA, Weinheim. (f) Schematic of the preparation of T-shaped gradient micropillars (TG) with calcium carbonate nanoparticles (CaCO_3_ NPs)/PDMS as materials by the PU mold method;^41^ Reprinted with permission. Copyright 2020 Wiley-VCH GmbH.

In Fig. 2c and d, PDMS is used to produce adhesive materials, but the preparation methods are different. Fig. 2d displays a PDMS film with a gecko-like multiscale structure derived from an aluminum oxide (AAO) template, which exhibits strong adhesion and a large contact area to almost any surface.^48^ As indicated in Fig. 2d, the PDMS microcolumn array is initially replicated from a surface-altered AAO template, followed by O_2_ plasma processing and APTES alteration. The silver nanoparticles previously prepared were adsorbed onto the APTES-modified PDMS tentacle arrays using electrostatic interactions and Au–N bonds. Subsequently, the resulting Au-modified array was immersed in a recently made Ag electroless deposition solution for several minutes. The resulting gecko-inspired nanotentacle surface-enhanced Raman spectroscopy (G-SERS) substrate enables effective target collection and a large contact area using a straightforward “press and peel” approach. Moreover, depositing silver nanoparticles in a densely packed three-dimensional nanotentacle array can create significant “hotspots”, guaranteeing a high amplification of SERS signals. As a result, the G-SERS substrate is better suited for microsampling and trace detection. Polydimethylsiloxane (PDMS) is a commonly utilized soft material that demonstrates remarkable biocompatibility, stability, high transparency, and ease of molding. This makes it an appealing material to be used in microfluidics, adaptable and wearable appliances. Therefore, reinforcing its structure is imperative to improve the mechanical properties of PDMS and to expand its potential applications. However, the low Young's modulus of PDMS makes it challenging to endure considerable external forces. Moreover, PDMS exhibits reduced toughness in comparison to natural rubber, which leads to insufficient resistance against crack propagation.

### 2.3. PEGDA-GelMA

In addition to microcolumn nanotentacle preparation, Fig. 2c fills the middle of the microcolumn with a hydrogel containing a micropore structure in an ordered fashion for cell culture.^47^ The micropore uses a precise sizing method for the cell sphere and facilitates nutrient supply to these cells. Fig. 2c displays the hierarchical hydrogel that has fully filled the gap between SiO_2_ nanoparticles and the personalized microcolumn array template through a bottom-up self-assembly technique. The prepared fractional hydrogels possess a structured arrangement of micropores and distributed nanopores at the micronano level. This structured arrangement was achieved through the template replication method. First, a PMMA template was replicated to obtain a uniform and smooth PDMS microarray. Then, an ethanol solution containing SiO_2_ nanoparticles was applied to the PDMS microarray. After ethanol evaporation, SiO_2_ nanoparticles self-assemble on the PDMS microarray. Subsequently, the gap between the nanoparticles and the microarray is fully occupied by a pregel composed of polyethylene glycol diacrylate (PEGDA) and methacrylate gelatin (GelMA) hydrogel. Once pregelled, UV photopolymerization takes place before the hydrogel is mechanically stripped. The nanoparticles were etched with hydrofluoric acid (HF) to obtain fractional hydrogels with ordered microstructures. SiO_2_ nanoparticles can self-assemble under van der Waals forces, hydrogen bonds, hydrophobic interactions, or other noncovalent forces, forming hexagonal close-packed structures on a two-dimensional (2D) substrate. By incorporating layered hydrogels into a multichannel concentration gradient microfluidic chip, a liver cancer chip was developed for efficient drug screening with excellent repeatability and accuracy. These attributes render the layered hydrogel system and its integrated cancer chip a top-notch platform for personalized medicine drug screening.

### 2.4. PU-gsmp composite rubber pad (GSMP)

The polymer's shape can undergo alterations under specific circumstances and remain fixed temporarily. The initial shape may be restored through external stimuli, including electricity, heat, light, or chemo induction. In comparison to PDMS, it offers enhanced shape variability, simpler processing, and better suitability for repetitive tearing in the application of human detection patches. Combining the characteristics of creeper adhesion and gecko adhesion, researchers were able to develop an artificial composite adhesive that exhibits firm and reversible adhesion on rough surfaces. Fig. 2e illustrates the artificial composite adhesive, which consists of polyurethane (PU) as a backing layer and graphene/shape memory polymer (GSMP) as a column array.^24^ A polyurethane-gsmp composite rubber pad (PU-GSMP) was prepared using the molding method. The GSMP mixture is poured onto the PDMS mold, replicated by the SU-8 mold. To remove any excess GSMP, a 0.7 mm thick UV-transparent PU film with high tensile strength (6.2 MPa) was pressed onto the PDMS mold. A uniform PU-GSMP is formed after curing at 120 °C for one hour. The GSMP microcolumns can switch between viscoelastic to glassy states due to graphene's photothermal effect. Benefiting from graphene's photothermal effect, the temperature of the GSMP microcolumn, and consequently, the energy storage modulus, can be controlled through UV irradiation.

### 2.5. Ink printing curing (IPC) technology

The model method offers benefits such as consistent product quality, convenient and straightforward preparation, and easy industrial scalability. However, manipulating the microstructure proves challenging, with a high rate of failure encountered during the preparation of complex structures. The array of microcolumns with T-shaped tips, as depicted in Fig. 2f, is produced utilizing ink printing curing (IPC) technology, wherein the modulus gradient is incorporated into the microcolumn *via* the gradient distribution of calcium carbonate nanoparticles within the microcolumn.^41^ The gradient of the modulus facilitates the formation of contact in the microcolumn and reduces perimeter stress while creating significant stress at the center of the contact area, resulting in enhanced adhesion and lateral friction. These design principles can be conveniently applied to other materials to achieve improved adhesion and lateral friction for soft materials.

## 3. Biomimetic ordered microstructures

### 3.1. Microcolumn

**Fig. 3:**
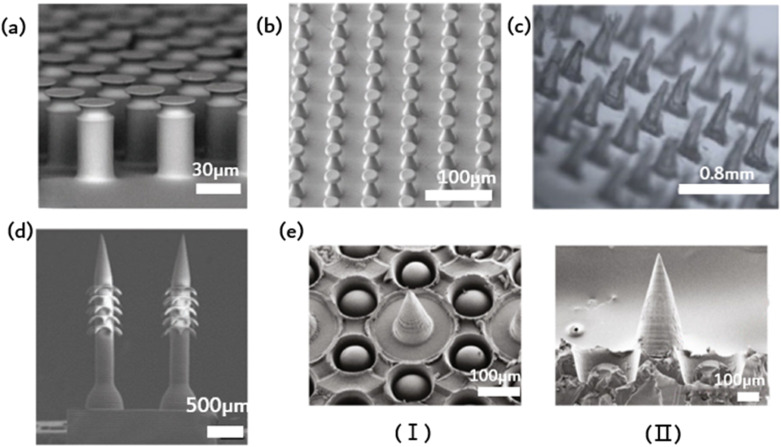
Ordered microstructure of bioinspired adhesion. (a) Schematic of the elastic flat punch cylinder array;^35^ Reprinted with permission. Copyright 2018 Wiley-VCH Verlag GmbH & Co. KGaA, Weinheim. (b) Schematic of soft elastomeric mushroom-like double re-entrant fibril arrays;^50^ Reprinted with permission. Copyright 2020 Wiley-VCH Verlag GmbH & Co. KGaA, Weinheim. (c) Schematic of the ordered array of microneedles;^31^ Reprinted with permission. Copyright 2023 Elsevier Ltd. (d) Schematic of microneedle array with rear-facing barbs;^51^ Reprinted with permission. Copyright 2020 Wiley-VCH Verlag GmbH & Co. KGaA, Weinheim. (e) Schematic of suction-cup-structured concave chambers combined with the MN structure;^52^ Reprinted with permission. Copyright 2020 Xiaoxuan Zhang *et al.* Exclusive Licensee Science and Technology Review Publishing House.

Contact, the universal phenomenon whereby objects attract each other, is exhibited by almost all types of matter. Biological evolution has led to the development of solutions for controlling adhesion, allowing organisms to attach to solids temporarily or permanently in various environments. This natural solution is suitable for standard environmental conditions, as well as high and low temperatures, variable humidity, and even underwater conditions. Bioinspired microstructural adhesives are a popular research topic worldwide. Fascinating examples of such microstructured adhesives can be found in the fibrous foot pad organs of insects, spiders and lizards in nature. These adhesives enable efficient movement on various substrates, allowing for quick attachment and separation within milliseconds. It is worth noting that a multitude of foot pads share remarkably similar morphology, characterized by elongated fibrillar structures exhibiting a length-to-diameter ratio between 10 and 80, which culminates in diverse end elements. The adhesion of toe pads in certain animals, such as geckos, can be partly explained *via* wagon interactions and capillary forces. Nonetheless, the specifics of contact geometry significantly impact bonding performance optimization. Dock beetles, such as the Gyrinidae family, demonstrate fibrils on their adhesive pads, featuring tip geometries from conical, scraper shapes through to mushroom-shaped. For every kind of fibril, there is significant variation in adhesion. The mushrooming tip achieves the highest pulling force and is identified by its gradual broadening toward the tip surface. Experimental evidence supports the effectiveness of the mushroom-shaped column (presented in Fig. 3a) that can increase pull out stress by up to ten times over the flat punch column.^35^ Unfortunately, due to their recessed geometry, these structures are not directly manufacturable. One option is to modify the apex of the column structure that was previously produced (in a straight form) by dipping it: drops of the liquid prepolymer are compressed between the cylinder and the substrate, creating a meniscus on every column. The prepolymers are subsequently cross-linked to produce a stable contact area. This approach is appropriate for a vast range of materials with nonvolatile prepolymers, *e.g.*, polydimethylsiloxanes or polyurethanes. The formation and ultimate shape of the mushroom tip are primarily determined by the wetting properties of the base and column surfaces, the size of the droplets used, and the degree of shrinkage during crosslinking. A significant discovery concerns the distribution of normal stress in the contact region. Flat punches in contact with inflexible substrates always display a stress singularity at their edges. Mushroom-shaped fibrils typically exhibit a significant reduction in the edge singularity size, alongside a concomitant increase in stress at the contact center. Notably, the stress distribution varies significantly with changes in tip geometry. Simulation findings suggest that a wide but slim mushroom flap promotes low edge stress. Some of these theoretical predictions have been qualitatively verified through experimentation. Mushroom-shaped fibrils typically exhibit a significant reduction in the size of the edge singularity, accompanied by a simultaneous increase in stress at the contact center. However, the actual stress distribution is strongly influenced by changes in tip geometry. The simulation results demonstrate that a wide but thin mushroom flap promotes low edge stress. Some of these theoretical predictions have been qualitatively verified through experimental means. The increase in the energy release rate during desorption changes based on the level of constraint. As the film thickness decreases (indicating a higher level of confinement), the detachment mechanism shifts from unstable growth of edge cracks to more stable growth of finger or central cracks. Furthermore, greater curvature results in increased tensile stress at the center.

### 3.2. Mushroom-like array

Fibrous adhesive pads offer robust adhesion to diverse surfaces and possess superhydrophobic characteristics. This water repellence can aid gecko footpads and their synthetic analogs in self-cleaning using the lotus leaf effect, thereby potentially preserving the functionality of the dry adhesion mechanism. Fig. 3b depicts an elastomer, stretchable mushroom-shaped fibrillar surface, which exhibits high adhesiveness, including liquids with low surface tension.^50^ Incorporate double recessed geometry into the bioinspired mushroom design of the fibrous dry adhesive to enhance adhesion strength. This approach maintains a smooth surface for fibril tips to promote optimal dry adhesion without requiring surface chemical modification. Compared to flat smooth surface control, this method yields up to five times the pulling force. The incorporation of double-concave protruding terminations onto fibrils does not impede their adhesive properties to the selected fibril material and geometry. In fact, it can aid in halting the propagation of cracks provoked near the fibril edge, thereby precluding adhesion failure. The researchers simulated the adhesion of a flat punch and fibril with a single reentrant tip shape and juxtaposed this with one possessing double-entrant fibrils. The findings demonstrate that, provided the cap diameter is identical, the adhesive force should be similar, and the effectiveness of double concave punches surpasses that of flat punches. Additionally, the double-concave headend shape's bending compliance rises compared to the single-concave scenario, which allows for more tension to extend through the headend. This could result in decreased shear durability of the adhesive, which, in turn, optimizes adhesive characteristics.

### 3.3. Microneedles

Common wound dressings frequently present suboptimal efficacy due to inadequate adhesion and failure to adapt to dynamic motion, ultimately resulting in delays in patient treatment. Inspired by mosquito mouthparts, an orderly array of microneedles is employed to enhance the adhesive capacity. Microneedles represent a drug delivery modality that falls between invasive therapy and adhesion, enabling precise medication administration while improving compliance.^9,53^ The specific structure of microneedles, being short and thin, greatly mitigates the risk of patient pain and infection during application.^31^ Additionally, the orderly arrangement of the MNs improves dressing adhesion strength and allows for precise drug delivery. However, using MNs for long-term drug delivery and microfluidic monitoring poses a challenge, as they must remain consistently attached to soft tissue for extended periods. This is primarily due to the standard microfabrication techniques applied in the MN fabrication process, resulting in a smooth and flat side profile of MN, causing weak tissue adhesion. In contrast, certain organisms in nature have developed impressive mechanisms for achieving strong tissue adhesion on a microscopic scale. Mosquitoes, for instance, feature hard, serrated axes on both sides of their feeding tubes, allowing for robust adherence and accurate anchoring during feeding. Endoparasitic nematodes affix themselves to the host's intestinal wall *via* an extendable proboscis, which mechanically interlocks with tissue for added reinforcement. The stingers of bees have miniature barbs that facilitate robust adhesion upon insertion. The stingers of bees have miniature barbs that facilitate robust adhesion upon insertion. Empirical evidence supports that barbed stingers show approximately 70 times greater adhesion strength than smooth stingers. In light of this adhesive property, a microneedle array with backward-facing barbs was devised (Fig. 3d).^51^ The regular array subsequently augmented adhesion. The dimensions of MNs with barbs might influence user comfort and the overall MN experience.

### 3.4. Composite structures

Imitating the sucker structure of an octopus, a cavity of sucker structure could encircle the barbed surface of each microneedle in addition to the existing barbs to increase adhesion, as depicted in Fig. 3e. This octopus tentacle imitation structure enhances the adhesion ability of microneedles in dry or humid environments. Scanning electron microscopy (SEM) images reveal that each cavity contains a dome-like projection. Notably, this structure resembles the suckers of an octopus, with a single bulge in each hole. The cross-linked hydrogel network that forms the basis of the made MNs arises from the covalent and noncovalent bonding of PDA and gelatin. Bioinspired multifunctional magnetic nanoparticles (MNs) have the potential to overcome the limitations of conventional techniques and serve as excellent candidates for versatile transdermal drug delivery systems.

The ordered arrangement of bionic microstructures can enhance adhesion, while the combination of different microstructures promotes the precise release of drugs from microfluidic patches and the benefits of enhanced adhesion. Additionally, the ordered structure facilitates the spontaneous drainage of liquids in microfluidics, promoting efficient separation and signal amplification. Capillary forces enable liquids such as wound exudates and skin interstitial fluid to flow spontaneously through the microfluidic channels designed on the ordered microstructure patch. Eventually, they reach the biomarker sensing detection area. Nevertheless, the existing biomimetic microstructures primarily enhance adhesion, and further research is needed on how to concurrently achieve drainage of the measured liquid and improve adhesion. Materials, preparation methods and applications of ordered structures was showed in Table 1.

**Table 1:** Materials, preparation methods and applications of ordered structures

Classification	Structure	Material	Processing method	Application
Microcolumn	Ordered microcolumn array^24,56,57^	Graphene/shape memory polymer (GSMP)	Mold casting method	Switchable strong adhesion
	T-shaped microcolumn array^41^	Calcium carbonate nanoparticles, PDMS	Template method; ink-print-curing (IPC) method	Super structural adhesive
	Hydrogel filled microcolumn array^47^	Polyethylene glycol diacrylate (PEGDA); methacrylate gelatin (GelMA)	Template copying method	Drug screening
Microneedle	Ordered microneedle array^51^	SF; PU; SP	Template copying method	Wound management
	Microneedle array with barbs^51^	poly(ethylene glycol) diacrylate (PEGDA 250)	4D printing	Enhanced tissue adhesion
Suction-shaped	Microneedle sucker complex array^52^	PEGDA-sodium alginate solution	Template copying method	Multifunctional transdermal drug delivery system
	Ordered sucker array^42^	Ecoflex liquid (silicone rubber); GelMA hydrogel	Template copying; mask guided lithography	Wound patch
	Cup array^52,55^	Polyurethane (PU)	Two-photon lithography, template replication	Underwater rough surface adhesion
Mushroom array	Ordered mushroom array^58^	PDMS	Microprojection stereolithography; soft template method	Elastic adhesion
	Ordered mushroom double recessed fibril array^24^	PDMS	Template method	Wet adhesion
Bristle array	Artificial gecko spatula^34^	Polyester microfiber; graphene		Precise particle assembly

## 4. Biomimetic adhesion based on ordered structures

The development of new materials through the replication or imitation of natural organisms' structure or function, commonly known as “bionics,” has been a longstanding practice. A new concept, however, has surfaced wherein characteristics from various biological species are combined to produce novel materials or devices with properties that surpass their biological prototypes or possess traits that are absent in either prototype. Combining the exceptional bonding components of both gecko and mussel allows for reversible bonding in wet and dry environments. Fibrous adhesives have been designed and constructed with various materials, some of which have even greater adhesion strength than gecko since the first evaluation of a single gecko bristle's adhesion (100 kPa) and the identification of the significant contribution of wagon force to gecko adhesion.

**Fig. 4:**
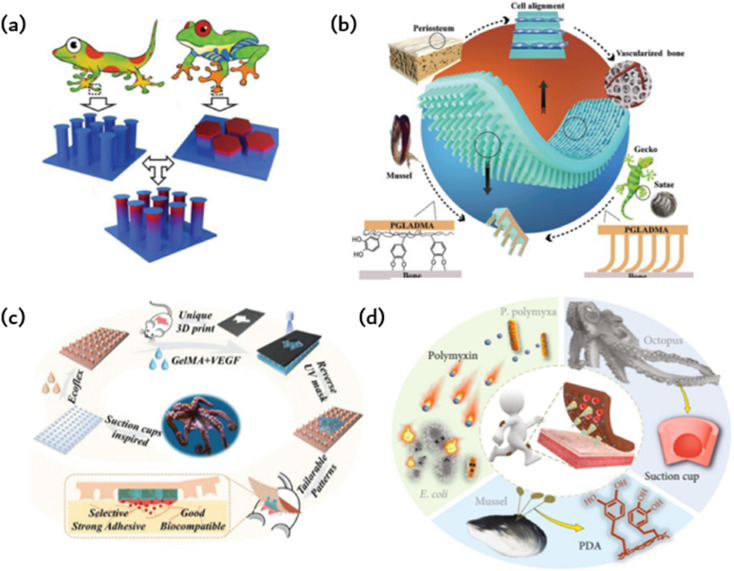
Bioinspired adhesion of ordered structures: (a) a gecko bristle inspired array of microcolumns with T-shaped tips and a tree frog-inspired microcolumn with gradient modulus form TG to provide stronger adhesion and friction;^41^ Reprinted with permission. Copyright 2020 Wiley-VCH GmbH. (b) Gecko-inspired fibrous bristle array for inner surface adhesion of artificial periosteum;^54^ Reprinted with permission. Copyright 2021 Wiley-VCH GmbH. (c) Octopus suckers mimic patches with strong and reversible adhesion to both dry and wet surfaces;^42^ Reprinted with permission. Copyright 2021 Wiley-VCH GmbH. (d) Microneedles (MNs) have a mussel-like polydopamine (PDA) hydrogel and are surrounded by a ring of cavities that mimic the suckers of octopuses;^52^ Reprinted with permission. Copyright 2020 Xiaoxuan Zhang *et al.* Exclusive Licensee Science and Technology Review Publishing House.

Drawing inspiration from biology, Fig. 4a imitates the structural adhesives that geckos use to adhere to surfaces. This is achieved by binding the adhesion organs of animals other than geckos, which enhances adhesion. A selection of animals possess adhesive organs with graded material features instead of graded fibers. The seta of the Ladybird beetle, for instance, has a gradient elastic modulus that gradually decreases from root to tip, ultimately promoting the formation of close contact and high structural stability. In contrast, the tree frog toe pad exhibits an inverse elastic modulus gradient that enables compliant contact with uneven or misaligned surfaces while maintaining high wear resistance. Fig. 4a illustrates the microcolumn array that combines the modulus gradient found in tree frogs with the T-shaped tip inspired by gecko bristles, thus forming the T-shaped gradient microcolumn (TG).^41^ For fibrils, empirical evidence supports the superiority of T-shaped tips as the optimal geometry for enhancing adhesion. Compared to hemispherical, blunt, and scraper tips, T-shaped tips facilitate better contact formation and ultimately produce a larger contact area. Furthermore, the well-designed overhang structure of the T-shaped microcolumn significantly diminishes stress on the contact perimeter, deters the initiation of cracks, and can even provide added vacuum suction to the separation interface. When the top of the T-shaped microcolumn is coated with a layer of softer material, adhesion can be further enhanced. The microcolumn array featuring T-shaped tips is produced through ink printing curing (IPC) technology, which entails a progressive modulus gradient inside the microcolumn that is achieved by means of a gradient distribution of calcium carbonate nanoparticles within it. The gradient of the modulus facilitates the formation of contact in the microcolumn, decreases stress around the contact, significantly amplifies the stress in the center of the contact area, and promotes a uniform formation of contact in the microcolumn. These effects lead to significant improvements in adhesion and transverse friction. The gradient modulus within the TG area enables contact formation and regulates stress at the separation interface, leading to 4.6-fold greater adhesion and 2.4-fold greater friction than the pure PDMS T-shaped microcolumn array. This research introduces innovative concepts for creating highly effective structural dry adhesives.

Due to the previously limited adhesion of artificial periosteum to bone defect sites, additional fixation is needed during implantation. This may also compromise their ability to inhibit the inward growth of soft tissue, which in turn prevents the formation of scar tissue in fractures. Taking inspiration from the gecko-mussel bonding mechanism, the Janus periosteum was proposed to imitate the structure and function of the natural periosteum by incorporating internal surface adhesion and external anatomical patterns (as shown in Fig. 4b).^54^ To achieve this, photocrosslinked polymers were utilized to replicate external anisotropic surfaces, structured micro slots for manipulation of cell fate and the assembly of gecko-inspired ordered fiber bristle arrays for inner surface adhesion. To improve its adhesion underwater, we coated the periosteal surface with mussel-stimulated poly(dopamine methyl-comethacrylate) (PDMH). The microbristle array and PDMH coating have a synergistic effect, resulting in the periosteum showing strong shear adhesion and normal adhesion in dry and wet conditions. Additionally, it has an efficient cellular regulatory effect that enhances simultaneous osteogenesis and angiogenesis without the need for growth factors.

The cup-shaped protrusions on an octopus, commonly referred to as suckers, possess the ability to adapt and effectively cling to various surfaces through physical adhesion. More precisely, the dome-shaped projection of the sucker facilitates extremely low internal pressure in relation to the ambient pressure, thereby producing robust and reversible adhesion characteristics for both dry and wet surfaces. Various synthetic adhesives featuring miniature suction cups have been developed and utilized for multiple applications in clean transfer systems, soft robotics, wearables, stimulus-responsive adhesives, and beyond. This is possible due to their advantageous properties. Taking inspiration from the suction cup structure of an octopus, researchers designed a patch using a silicone rubber (Ecoflex) membrane (as shown in Fig. 4b).^42^ This membrane features a microstructure similar to a suction cup, allowing it to adhere to normal skin. The patch also includes a biocompatible GelMA hydrogel to directly contact and treat the injured area. Owing to the microstructure of the sucker on the Ecoflex membrane, the patch can strongly adhere to both dry and wet surfaces. Moreover, GelMA presents significant similarity to the extracellular matrix, ensuring the excellent biocompatibility of the patch. Additionally, the GelMA hydrogel was supplemented with vascular endothelial growth factor (VEGF) to further expedite the healing process. The GelMA hydrogels are shaped according to the random geometry of individual wound areas during the manufacturing process using an ultraviolet (UV) mask with customizable patterns. This integration of the opposite properties of adhesion and antiadhesion results in a single patch film. For simple column arrays and flat patches, adhesion is solely due to capillary force, whereas octopus sucker microstructure patches exhibit stronger adhesion.

Hydrogels are utilized to deliver medications to injured regions and improve adhesion to a certain degree. Nevertheless, the coherence of the hydrogels does not promote the detection of microfluidic chips. A composite, structured, microneedle and cup patch is introduced (as shown in Fig. 4d), as most hydrogel microneedles do not conform well to the skin and detach easily.^52^ They rely heavily on aids such as medical tape for ideal penetration and fixation. The limited adhesive quality of hydrogel microneedles considerably restricts their practical usability, particularly in areas that undergo extensive motion. Various organisms in nature possess remarkable adhesion capabilities due to the molecular attraction or microstructure of their surfaces. As a classic illustration, mussels can firmly attach themselves to almost any surface *via* noncovalent and covalent chemical interactions between the adhesive proteins of the mussel byssus and the surface. Octopuses, in contrast, can attach themselves to dry or wet objects due to their suckers and dome-shaped internal projections. Graded microneedles with multifunctional adhesion were developed, inspired by the adhesion mechanism of octopuses and mussel tentacles. A polydopamine hydrogel is used as a needle base, whereas each microneedle is encompassed by a suction cup cavity structure. The resulting microneedles can adhere well to the skin, maintain strong adhesion in dry and wet conditions, and demonstrate self-repair after splitting into two parts. Interestingly, every microneedle is encircled by a ring of concave chambers with dome-shaped protrusions situated inside. This design, inspired by the tentacles of an octopus, enhances adhesion, resulting in microneedles that can now adhere in dry or wet environments. The negative mold comprises a systematic grouping of conical cavities, each encompassed by six protrusions. It should be noted that this design can enhance protrusion density, improving the suction and adhesion of the replicated microneedles. To create MNs with the desired structure and bonding properties, a PEGDA-sodium alginate solution is added to the negative mold as a MN tip, and a DA-gelatin mixture is added to the MN substrate and cured successively.

The first adhesives used by humans were derived from natural sources, such as animal gelatin and starch. However, these biological adhesives had poor adhesion and were only suitable for use on limited surfaces. In the last century, the polymer industry has rapidly developed to compensate for the shortage of natural adhesives. However, simple adhesives cannot meet the requirements of strong adhesion and switchable adhesion in medical applications. The observation that geckos can crawl on vertical surfaces has provided a solution to this problem. Therefore, the aforementioned studies simulate the surface microstructure of adherent organisms. Simultaneously, the incorporation of biomimetic microstructures enhances the performance of microfluidic chips.

## 5. Microfluidic based on ordered structures

Bionic structures, such as lotus leaves with self-cleaning function, gecko feet with directional adsorption ability, butterfly wings with special optical properties, shark skin with flow drag reduction function, and super-slippery pitcher leaves, will help break the technical bottleneck of microfluidic. Bionic microfluidics has a strong interdisciplinary nature that can inspire new designs and research for microfluidic applications. This will undoubtedly promote the functional development of microfluidics and provide new impetus for its future development. Currently, bionic structures for microfluidic systems mainly focus on four aspects: promoting fluid self-flow, anti-pollution devices, biomimetic microfluidic pump and improved wear comfort,. The following will focus on these four representative aspects.

### 5.1. Promoting fluid self-flow

**Fig. 5:**
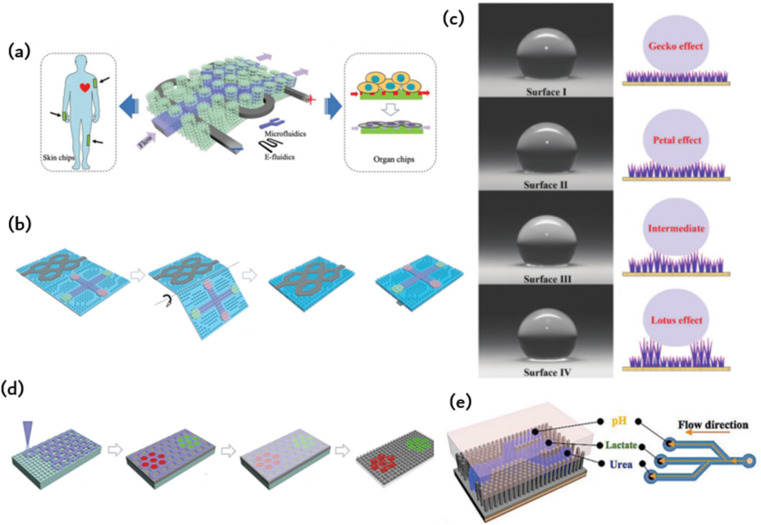
Microfluidic chips with ordered microstructures: (a) meta-structured membranes (MSMs) as substrates for fabricating integrated microfluidics and electronics;^59^ Reprinted with permission. Copyright 2019 Wiley-VCH Verlag GmbH & Co. KGaA, Weinheim. (b) Microfluidic channels and electro circuits were first patterned on the ordered MN and then origami to form a double-side integrated chip;^60^ Reprinted with permission. Copyright 2020 Wiley-VCH GmbH. (c) Schematic diagram of hydrophobic surface based on bioinspired fabrication;^26^ Reprinted with permission. Copyright 2022 Wiley-VCH GmbH. (d) The photonic crystal pattern is assembled by elastic copolymer nanoparticles in an ordered microcolumn array to enable electronic skin biochemical detection;^57^ Reprinted with permission. Copyright 2022 Wiley-VCH GmbH. (e) Schematic diagram of constructing microfluidic channels in an ordered nanotentacle array;^22^ Reprinted with permission. Copyright 2018 Wiley-VCH Verlag GmbH & Co. KGaA, Weinheim.

The detection of potassium (K+), sodium (Na+), lactic acid, and glucose in sweat has a wide range of applications in various fields. These include monitoring the course and treatment of chronic diseases such as diabetes and kidney disease, evaluating exercise performance and physical exertion, and conducting pharmacokinetic evaluation in drug development and clinical trials. Sweat analysis is a valuable tool for monitoring the daily physiological indicators of individuals in key positions, such as pilots and astronauts. Wearable forms of sweat detection dominate in such scenarios due to their ability to provide real-time, long-term, and reliable monitoring. However, maintaining fresh sweat in the system is crucial for continuous real-time monitoring and high-precision results in the field. Previous wearable sweat detection systems typically use hydrophilic and porous materials to collect sweat. However, absorbent materials can be difficult to discharge the collected sweat effectively, resulting in reduced detection accuracy and making long-term sustainable use challenging. Currently, Gao *et al.* have proposed the tree frog's toe pad is combined with the micro/nanostructure of the gecko foot to simulate the construction of the sensitive skin bonding layer.^22^ The resulting ordered column arrays are prevalent in microfluidic chip technology, capacitive electrodes, and hyperadhesive materials. Capillary forces among microcolumn arrays contribute to the flow of liquids without a pump, and pyramid arrays can effectively enhance the interaction between materials, thus boosting efficiency in capacitance and triboelectricity. Additionally, ordered micron structures offer several benefits, and ordered nanostructures, such as photonic crystals, are widespread in nature, as seen in the wings of butterflies and peacock tails. Inspired by nature, this study proposes a meta-structural membrane (MSM) as a substrate for fabricating integrated microfluidic and electronic devices (refer to Fig. 5a). These flexible and independent MSM produce micropatterned PDMS templates on photonic crystals of elastic plastic copolymer nanoparticles. This production method allows for self-assembly, resulting in an MSM with an integrated ordered micron and nanostructures. The MSM exhibits spontaneous liquid transfer, fluorescence enhancement, and intimate skin contact. A patterned mold is used in the manufacture of a design-pattern MSM through the assembly of polymer nanoparticles. By using these patterns as microfluidic channels and circuits, a highly integrated and complex electric microfluidic is formed on one slice of MSM. These MSMs may be utilized as on-chip sensors on the skin, for mixed biochemical–physiological monitoring sensing in the human body, and as organ chips for cell culture and metabolite analysis under drug therapy. The skin-mounted chip sensitively detects movement frequency, micromovement (pulse beat) and cardiac biomarkers (myocardial troponin I (cTnI), creatine kinase isoenzyme (CKMB), and myoglobin (Myo)) for effective human cardiac care. Moreover, the organ chip facilitates human cancer cell culture, reactive oxygen sensing and identification of lactic acid under anticancer drug treatment. These diverse applications illustrate the potential of MSM in creating highly integrated devices that possess multiple functions.

### 5.2. Biomimetic microfluidic pump

The micropump is responsible for driving the fluid flow on the microfluidic chip and is considered the power and core component of the microfluidic system. The biomimetic system enables the flow and mixing of liquids through external electrostatic fields, magnetic fields, air pressure, or light stimulation. This system is significant for microfluidic research and establishing a model system for basic research of fluid dynamics in biological samples.

Hydrogel patches are frequently employed to accelerate wound healing owing to their drug-loading capability and flexibility, whereas microchips with multiple integrated microchannels on their surface can gauge wound conditions through the extraction of wound secretions and the analysis of specific biomarkers.^60^ In addition, Fig. 5b displays a well-ordered microarray with controllable size and capillary properties, making it suitable for microfluidic applications. Due to capillary forces, the microfluidics diffuse spontaneously along the microfluidics channel and come into full contact with the IO PC structure in the detection region. Detecting the fluorescence signal in the detected area allows for the relative content of specific biomarkers in wound secretions to be determined. Spontaneous fluid flow in microarrays presents excellent application prospects in various research fields. Permeability is a crucial factor in the design and application of these arrays. The patterned tip of the smart patch integrates with the IO PC structure to create multiple porous structures, thus improving its drug loading capacity. Moreover, a microelectronic circuit is included to detect the wound area's motion state. This highly integrated smart patch demonstrates exceptional performance in controlled drug release, biochemical analysis, motion sensing (finger, wrist, and elbow bending), and wound healing. These distinctive features indicate that the intelligent patch shows potential for wound management and could present promising prospects in various analogous biomedical spheres.

### 5.3. Anti-pollution device

Contamination refers to the non-specific adsorption of non-target substances, such as proteins and cells, on the surface of the device. This can reduce the reliability and service life of the device. Microfluidic chips also face pollution issues due to their miniaturisation, which results in a high specific surface area. Consequently, more areas on the channel are susceptible to pollution. In order to apply microfluidic technology in practical settings such as biomedicine, energy-saving systems, and high-efficiency material separation, it is essential to reduce or eliminate pollution of the microfluidic chip.

Protein adsorption is affected by various factors, including hydrophobicity, electrostatic adsorption, van der Waals force, and hydrogen bonding force. Proteins have a tendency to adsorb on non-polarized, high-tension, and charged surfaces, which can lead to contamination of materials or devices. Surface structural properties, such as roughness and infiltration, also have a significant impact on protein and cell adsorption. To address the issue of device pollution, there are generally two main approaches. The first step in improving the anti-fouling performance of microchannels is to select or prepare materials with low surface energy. Polytetrafluoroethylene materials can be used to achieve acid–base resistance and anti-biological pollution ability in microchannels. Another option is to create 3D micro-nano structures to reduce surface energy. To enhance the anti-fouling performance of the microchannel, the inner surface of the microchannel material can be physically and chemically modified. Biomimetic microfluidics primarily improve anti-pollution performance by utilising biomimetic materials and surface modification methods.

Superhydrophobic (SHPO) surfaces are widely accepted due to their water contact angle (CA) of over 150°. This property has been applied in antifouling, oil/water separation, and heat transfer. In the past two decades, researchers have proposed various bionic SHPO surfaces inspired by natural phenomena such as lotus leaves, gecko feet, rose petals, butterfly wings, and springtails.^26^ The literature suggests that surfaces resembling gecko skin, which have single-scale nanotextures, are composed of closely packed and vertically aligned nanotubes, nanowires, or nanorods. Furthermore, surfaces with petal and lotus effects have double-scale nano/micron textures. Superhydrophobic surfaces have been extensively employed for self-cleaning, anti-fouling, and separation. Gecko and petal-shaped surfaces show promise in applications requiring high adhesion, such as microfluidic control and drug release. Furthermore, gecko surfaces display velocity-sensitive adhesion, making them suitable for use in areas where high-speed droplets are employed and strong adhesion is necessary.

Xu *et al.* proposed a self-cleaning system for gecko spatulas. They were inspired by the scraper structure of geckos and its dynamic effects.^34^ To create artificial bristles, they used synthetic polyester microfibers with a diameter of 10 mm. Each fiber was 150 mm in length and was cut to form a micropad at its tip. The micropad was then bonded to the atomic force microscopy (AFM) cantilever (refer to Fig. 5c). Three folded graphene layers, each 5 nm thick, were then glued layer by layer onto the micropad. Previous studies have shown that graphene produces superior adhesion on various surfaces. A pleated graphene layer on the pad, similar to a gecko scraper on the bristles, can significantly improve the adhesion of artificial bristles by increasing surface compliance and contact area. Graphene layers can produce reversible and adjustable adhesive forces, making them suitable for various applications such as self-cleaning and manipulation of small objects in air. Xu *et al.* successfully manipulated microspheres on a variety of substrates using artificial bristles attached to atomic force microscopy (AFM) probes as micromanipulators underwater. Unlike traditional adhesives like Scotch tape, this technology offers significant advantages. Furthermore, it can accurately arrange particles that are only a few microns in size in a patterned manner, as illustrated in Fig. 5b. The research shows that the self-cleaning and micromanipulation capabilities of the gecko scraper nanomats are strong and effective in synthetic bioinspired adhesives. These results indicate that the gecko scraper nanomats' distinctive dynamic effects can be applied to a variety of applications.

### 5.4. Improved wear comfort

The use of microfluidic technology in wearable devices designed for ergonomic comfort must ensure long-term comfort. Taking inspiration from gecko feet, researchers created a microcolumn array with adhesive properties. This design was then enhanced with mussel-stimulated polydopamine (PDA) to further increase adhesion. By using a suspension of P (MMA-BA) elastic copolymer nanoparticles with various sized particles, which are deposited in a regionalized photonic crystal pattern on the electronic skin, multiple biomarkers can be detected simultaneously. Electronic skin has the ability to carry out biochemical tests on injured and healthy skin. Fig. 5d depicts an electronic skin that possesses high stretchability and remarkable strain sensing capabilities, which permits it to identify biomarkers.^57^ Electronic skin can be useful in improving robotic precision during tasks such as grasping and positioning and augmenting human–computer interaction through the perception of human touch. The electronic skin can also be affixed to a soft robot for sample collection in restricted or hazardous locations, where humans cannot maneuver, thus offering immediate feedback during the procedure. An alternative application involves attaching the skin to animal epidermis to monitor health and improve the diagnosis of metabolic diseases and wound inflammation. This study offers a fresh approach to improve the efficiency of electronic skin by integrating bionic structures and developing multifunctional electronic skin.

Fig. 5e depicts a microfluidic system equipped with a polyvinylidene fluoride (PVDF)-based piezoelectric nanogenerator and nanotentacle skin inspired by gecko.^22^ The 3D nanotentacle array paper made from nitrocellulose (NC) is both flexible and self-contained. The system demonstrates the simultaneous sensing of pH, urea, and lactic acid levels in perspiration. Lithographic patterns are imprinted on the NC paper with stainless steel impressions to create microfluidic channels with three branches for perspiration diversion. The AAO template is infused with NC precursors and then etched to acquire the nanotentacle setup. Once the liquid is entirely extracted, the nanocolumns self-organize to create numerous configurations. The NC nanoantenna paper subsequently carries the pattern through a stainless-steel impression process. The raised structure amalgamates the nanotentacles, generating hydrophilic and hydrophobic areas on the substrate to create microfluidic pathways on the nanocolumns. The nanoantennae inspired by geckos are also capable of conforming to rough skin, thereby improving the PENG signal. The plasticity of the 3D tentacle structure of NC paper enables the material to establish a diverse array of interactions with virtually any surface, thereby allowing for conformal contact with rough skin surfaces. For the creation of microfluidic channels on NC paper possessing abundant micro/nanostructures, NC nanotentacles were patterned employing impressions.

The arrangement of the microstructure can facilitate the self-flow of liquid in the microfluidic chip, with some structures serving as a microfluidic pump. To address the issue of high susceptibility to pollution due to the large specific surface area of the microfluidic chip, an imitation hydrophobic surface is effective in self-cleaning. The use of ordered microstructure achieves strong and switchable adhesion, effectively addressing the issues of weak adhesion and discomfort caused by tearing of wearable microfluidic chips.

## 6. Integration of ordered structures-based adhesion and microfluidics

Inspired by bioadhesion in nature, numerous researchers have developed a method to enhance adhesion by engaging with the microstructure of a surface. This microstructure-driven adhesion differs from prior biadhesive films and enables long-term wear of microfluidic chips, leading to the concept of artificial skin. An integrated microelectronic circuit detects the movement of the wounded area. The smart dressing is highly integrated and displays exceptional abilities in controlled drug release, biochemical analysis, and motion sensing (including finger, wrist, and elbow movements), as well as wound healing. These particular qualities indicate that smart origami microneedle dressings hold much promise for the management of wounds and offer substantial potential in various biomedical fields. This electronic skin can enhance robot precision in tasks such as object manipulation and increase the degree of human–computer interaction by enabling robots to perceive human touch. Additionally, the electronic skin can be attached to a soft robot to obtain samples from narrow or dangerous locations inaccessible to humans, enabling real-time feedback during the process. Alternatively, the sensor should be affixed to animal skin to monitor health and aid in diagnosing metabolic diseases and wound inflammation. Its conformal, firm, and comfortable adhesion properties are displayed when applied to the skin surface. Wearable skin sensors are valuable for personalized healthcare. The adhesion between the flexible sensor and the skin surface is crucial to achieving accurate, dependable, and steady signals. The bond between the pliable sensor and the skin surface is paramount in obtaining precise, dependable, and consistent signals.

### 6.1. Wound healing

**Fig. 6:**
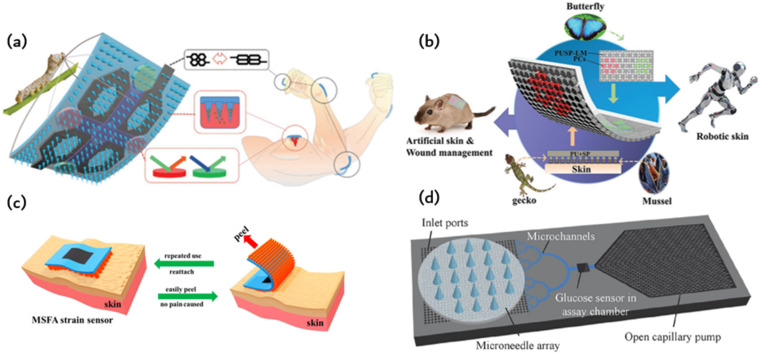
Application of ordered bioinspired adhesion microfluidics: (a) intelligent origami silk fibroin microneedle-structured dressing (i-SMD) for biochemical sensing, motion monitoring and wound healing;^60^ Reprinted with permission. Copyright 2020 Wiley-VCH GmbH. (b) Bionic electronic skin attached to the skin for motion monitoring and biochemical detection;^57^ Reprinted with permission. Copyright 2022 Wiley-VCH GmbH. (c) The repastable microstructured fibroin adhesive (MSFA) strain sensor;^61^ Reprinted with permission. Copyright 2020 American Chemical Society. (d) Schematic diagram of a microfluidic chip for continuous sampling;^62^ Reprinted with permission. Copyright Springer Nature.

Fig. 6a illustrates a smart origami dressing with a silk fibroin microneedle structure, which is combined with an IOPC structure, microfluidic channels and microelectronic circuits to promote wound healing while monitoring the process of wound recovery.^60^ The silk fibroin (SF)-based MNs are patterned and manufactured using a negative polydimethylsiloxane (PDMS) mold created by a laser engraving machine. A cross-shaped microfluidic channel has been patterned onto a clever origami microneedle dressing, with four detection areas of the channel featuring IOPC structures. Upon attaching the smart origami microneedle dressing to the wound area, the fluid can diffuse spontaneously along the microfluidic channel due to capillary forces, thus fully engaging the IOPC structure in the test area. By detecting the fluorescence signal in the affected area, the relative content of specific biomarkers in wound secretions can be determined objectively. The tip of the patterned smart dressing integrates with the IOPC structure, creating several porous structures that improve the drug loading capacity of the smart origami microne dressing.

### 6.2. Motion monitoring

Fig. 6b presents an electronic skin that has exceptional strain sensing and high stretchability and is capable of detecting biomarkers.^57^ The design was inspired by the adhesive capabilities of gecko feet, leading to the development of a microcolumn array that was further enhanced by incorporating mussel-stimulated polydopamine (PDA) for improved adhesion. A suspension of P (MMA-BA) elastic copolymer nanoparticles with varying particle sizes is employed to create a regionalized photonic crystal pattern on electronic skin, enabling the simultaneous detection of multiple biomarkers. This study utilizes electronic skin for the biochemical detection of both normal and injured skin. Fig. 6c depicts a fibroin binder (MSFA) that possesses a microcolumn structure on a PDMS substrate.^61^ Microstructured fibroin adhesives (MSFA) demonstrate dependable adhesion on the skin's surface, even in cases of moisture or dampness, and facilitate facile removal from the skin without causing significant discomfort.

Fig. 6c depicts a fibroin binder (MSFA) that possesses a microcolumn structure on a PDMS substrate.Microstructured fibroin adhesives (MSFA) demonstrate dependable adhesion on the skin's surface, even in cases of moisture or dampness, and facilitate facile removal from the skin without causing significant discomfort.

### 6.3. Drug delivery

Medical methods that use wearable devices to administer drugs through the skin, such as drug patches, have a long history. However, the biggest disadvantage of these patch delivery technologies is the difficulty in controlling the dose and the short duration of use. The initial drug dose is often too high, and with extended application time, the administration dose quickly decreases. This constant change in drug dose is not conducive to controlling the corresponding disease. Researchers have proposed using wearable microfluidic chip technology for drug delivery. However, poor adhesion of microfluidic chips and resulting poor user experience have been major obstacles to putting this idea into practice. The emergence of bionic adhesion technology provides a new method for wearable microfluidic chips. This method utilises a wearable device for storing drugs, which is combined with microneedles or a microneedle array to puncture the skin and deliver medicine. The precise control of the shape and length of microneedles allows them to penetrate the skin surface without touching the nerve layer, resulting in painless puncture and accurate drug delivery. The combination of a microneedle array and microfluidic chip enables medicine to be delivered in the form of micron or even nanoparticle encapsulation. Fig. 2 shows the various types of microneedles that can be used for puncture and drug delivery purposes. These microneedles are made of different materials, including metal, silicon, and polymer. The processing methods used to create these microneedles are inherited and developed from the processing methods of micro-electromechanical systems (MEMS) and microfluidic systems. Processing hollow-shaped microneedle arrays presents challenges, so solid microneedle arrays can be used for drug application and slow release of medication. Processing hollow-shaped microneedle arrays presents challenges, so solid microneedle arrays can be used for drug application and slow release of medication. Biodegradable microneedle arrays are also an option.

Fig. 6d shows a basic wearable microfluidic drug delivery device that immobilises the drug in the form of a nanoparticle in a gel. The gel is loaded onto a soft microfluidic chip substrate and covered with a layer of microneedle arrays. When medication is required, a micro-needle is inserted into the skin by bending the finger. The gel is then pressed to release drug-containing nanoparticles for delivery. According to the authors, this wearable microfluidic drug delivery device can release anti-inflammatory and pain relief drugs, as well as blood glucose detection signals. It can also precisely release insulin to regulate blood sugar levels.

The primary use of ordered microfluidic adhesive chips is for wound healing detection, motion detection, and microfluidic chip delivery. The bionic adhesion structure provides strong and switchable adhesion, enabling real-time assessment of wound status and movement information by analyzing wound secretion data and movement data through the microfluidic chip.

## 7. Conclusion and outlook

This paper provides an overview of the materials and fabrication techniques used to create ordered microstructures. Additionally, it explores bionic ordered adhesion and microfluidics within such structures, which possess features such as spontaneous liquid drainage, streamlined separation, and signal amplification. Consequently, this study proposes integrating ordered biomimetic adhesion microfluidics. It offers strong adhesion and limited real-time monitoring of biochemical signals. However, some challenges need to be addressed in this promising and attractive research field.

On the one hand, the impact of adhesion must be construed in terms of microfluidic analysis and detection outcomes. During the transition of the adhesive patch from its “hyperadhesive” state to the “easily peeled” state on the skin, the microordered structure undergoes changes that impact the stability of the microfluidic channel. Subsequently, this affects the accuracy and reproducibility of the analysis results.

Additionally, microfluidic channels and solution infiltration have an impact on adhesion. For instance, the microfluidic patch adhered to the skin surface facilitates health monitoring of the wearer by detecting sweat, glucose, and biological factors in wounds. However, the adhesion failure at the interface and diffusion of the bulk adhesive results in a decrease in adhesive strength.

The microfluidic channel's high specific surface area makes it more vulnerable to external pollution than other materials. The introduction of the bionic ordered structure adds new details to its complex internal structure, further increasing its susceptibility to external liquid pollution. Therefore, designing a contamination-free microfluidic chip is crucial for enhancing its function and adhesion. Currently, some scholars have proposed using a bionic self-cleaning biological surface to prevent pollution in microfluidic chips.

Third, the processing methods and material selection used in biomimetic adhesion microfluidics with ordered structures are critical to their performance. As future manufacturing methods demand biocompatible, cost-effective materials, it is important to simplify the process of moving the research from the laboratory to production. There are already some studies that have simplified the preparation process, but many challenges remain in commercial-scale manufacturing.

In summary, the integration of ordered structures, biomimetic adhesion, and microfluidics has emerged as a thriving area of research with significant interdisciplinary applications. It is anticipated that as the processing of ordered structures for adhesion and microfluidics advances, there will be increased participation from researchers across various fields, facilitating the development of adhesive-structured microfluidics into a more dependable technology for industrial use. Furthermore, we anticipate that adherent structured microfluidics will undoubtedly offer a novel solution for health monitoring and therapy in the future.

## Author contributions

Conceptualization, writing—original draft preparation, literature search, figures, Meng Wei and Qian Zhou; investigation, Meng Wei; project administration, study design, data collection, funding acquisition, Xiaoming Ma and Bingbing Gao.

## Conflicts of interest

The authors declare that they have no known competing financial interests or personal relationships that could have appeared to influence the work reported in this paper.

## Supplementary Material
